# Ultrasonic bone removal from the ossicular chain affects cochlear structure and function

**DOI:** 10.1186/s40463-021-00491-4

**Published:** 2021-04-02

**Authors:** Jennifer M. Siu, Jaina Negandhi, Robert V. Harrison, Nikolaus E. Wolter, Adrian James

**Affiliations:** 1 Department of Otolaryngology - Head & Neck Surgery, University of Toronto, Toronto, Canada; 2 Program in Neuroscience and Mental Health, Hospital for Sick Children, Toronto, Canada; 3 Department of Otolaryngology, Hospital for Sick Children, University of Toronto, 555 University Avenue, Room 6133, Burton Wing, Toronto, ON M5G 1X8 Canada

**Keywords:** Ultrasonic bone removal, Piezosurgery, Safety

## Abstract

**Introduction:**

Ultrasonic bone removal devices (UBD) are capable of cutting through bony tissue without injury to adjacent soft tissue. The feasibility and safety of using this technology for removal of bone from an intact ossicular chain (as might be required for otosclerosis or congenital fixation) was investigated in an animal model.

**Methods:**

This was a prospective animal study conducted on seven anesthetised adult chinchillas. An UBD was used to remove bone from the malleus head in situ*.* Pre and post-operative distortion product otoacoustic emission (DPOAE) levels and auditory brainstem response (ABR) thresholds were recorded. Scanning electron microscopy (SEM) was used to assess cochlear haircell integrity.

**Results:**

Precise removal of a small quantity of bone from the malleus head was achieved by a 30s application of UBD without disruption of the ossicular chain or tympanic membrane. DPOAEs became undetectable after the intervention with signal-to-noise ratios (SNR) < 5 dB SPL in all ears. Furthermore, ABR thresholds were elevated > 85 dB SPL in 13 ears. SEM showed significant disruption of structural integrity of the organ of Corti, specifically loss and damage of outer haircells.

**Conclusions:**

Although UBD can be used to reshape an ossicle without middle ear injury, prolonged contact with the ossicular chain can cause structural and functional injury to the cochlea. Extensive cochlea pathology was found, but we did not investigate for recovery from any temporary threshold shift. In the authors’ opinion, further study should be undertaken before consideration is given to use of the device for release of ossicular fixation.

**Graphical abstract:**

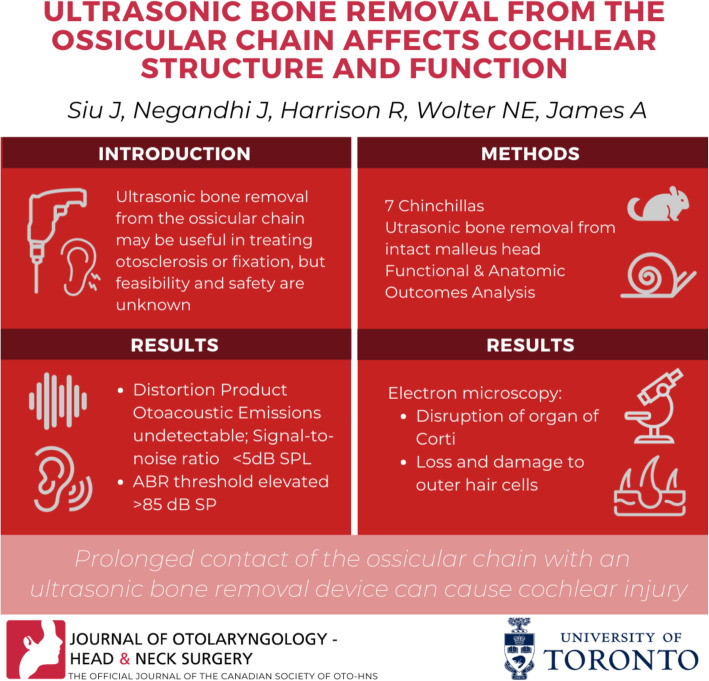

## Introduction

Ultrasonic bone removal devices (UBD) are capable of making precise cuts through bone without injury to adjacent soft tissue [1–6]. Originally used in oral surgery, the technology has been adopted in various other surgical fields including neurosurgery, spine surgery, reconstructive surgery, hand and wrist surgery [7, 8]. These qualities are potentially advantageous in otologic surgery for example to remove bone adjacent to the facial nerve. The opportunity for bone cutting, irrigation, and in some devices suction, with a single handpiece has also favoured its adoption in endoscopic ear surgery.

Despite several reports on the relative safety of the device, conclusions are still equivocal. The UBD generates heat and produces intense, ultra-high-frequency sound which has the potential to cause damage to the cochlea and inner ear when used in close proximity [9]. In some studies, no inner ear changes were reported when using the device for stapedotomy and other middle ear surgeries [5, 6], while other studies describe high-frequency hearing loss and postoperative vertigo from similar procedures using the same device [10, 11]. These studies in various human and animal settings have all concluded that more research is required for a more comprehensive understanding of its safety in otologic surgery [5, 6, 10–12].

The objectives of this study are: to determine the effects of an UBD on the functional integrity of the cochlea as measured by otoacoustic emission (OAE) and auditory brainstem response (ABR) threshold recording and secondly, to determine its effect on the structural and anatomic integrity of the cochlea hair cells using scanning electron microscopy (SEM). An animal model was used to evaluate the effects of UBD application to an intact ossicular chain, such as might be performed for release of congenital ossicular fixation or otosclerosis.

## Materials and methods

### Animals

Adult chinchillas *(Chinchilla lanigera)* were chosen as the animal model of choice in this study because of the easy surgical access to the middle-ear structures through a large bulla cavity. This animal also has a relatively low frequency range of hearing, closer to the human hearing range than other common laboratory species. Our laboratory protocol for surgical access with this animal has been documented previously [13]. Prior to surgery, all animals were anesthetized with intraperitoneal ketamine (15 mg/kg) and xylazine (2.5 mg/kg). Additional one-half doses of anesthetic were administered hourly and as required based on muscle tone and respiratory pattern. All testing and surgeries were performed within a sound-attenuating chamber. All procedures and protocols were approved by the Ethics and Animal Care Committee at the Hospital for Sick Children, Toronto, following the guidelines of the Canadian Council on Animal Care (CCAC).

### Baseline ABR and DPOAE threshold determination

ABRs and DPOAEs were evaluated in all subjects before and after experimental manipulation to establish initial normal hearing thresholds and any changes caused by surgical interventions. The signal to noise ratio of DPOAE was recorded from each ear (Echoport, Otodynamics Ltd., Herts UK). The distortion product was recorded at 2f_1_ – f_2_, from primary tones at an f_2_ of 1, 2, 4 and 8 kHz (f_2_ /f_1_ = 1.22) and intensity levels of L_1_ = 65 dB SPL and L_2_ = 55 dB SPL. For ABR testing, electrodes were placed in a standard mastoid/bulla-to-vertex configuration. ABR thresholds were obtained to tone pips at 2, 4, and 8 kHz. Stimuli were presenting using ER2 electrostatic speakers fitted with a 1.5 cm polyethylene tube and a conforming cuff positioned in the ear canal. Tone pips were presented at 24–32/s. Stimuli were presented over a 15-90 dB SPL range of intensity in 5-10 dB steps. ABR signals were amplified, filtered (100 Hz to 3 kHz) and averaged (1000 sweeps; Intelligent Hearing Systems, Miami USA). Threshold responses were determined by visual inspection of ABR waveforms. Only animals with normal hearing (thresholds below 30 dB SPL; 2–8 kHz) were included.

### Surgical approach to middle ear and initial testing

The UBD used in this study (Piezosurgery Medical Device; Mectron Medical Technology, Carasco, Italy) has various settings to adjust for the bone thickness and density of the tissue being drilled. A “Cortical” setting for high-density bone mineralized bone; “Cancellous Medium” for lamellar medium-density cancellous bone, “Cancellous Low” for lamellar low-density cancellous bone; and “Delicate Anatomy” for bone thickness less than 1 mm and close to neurologic soft tissue [12]. For this study we chose to perform the drilling with the ultrasonic bone removal device using a 1.2 mm tip and the instrument set at Delicate Anatomy mode, power level 4 with continuous vibration (vibrating speed of 20,000 rpm).

**Fig. 1:**
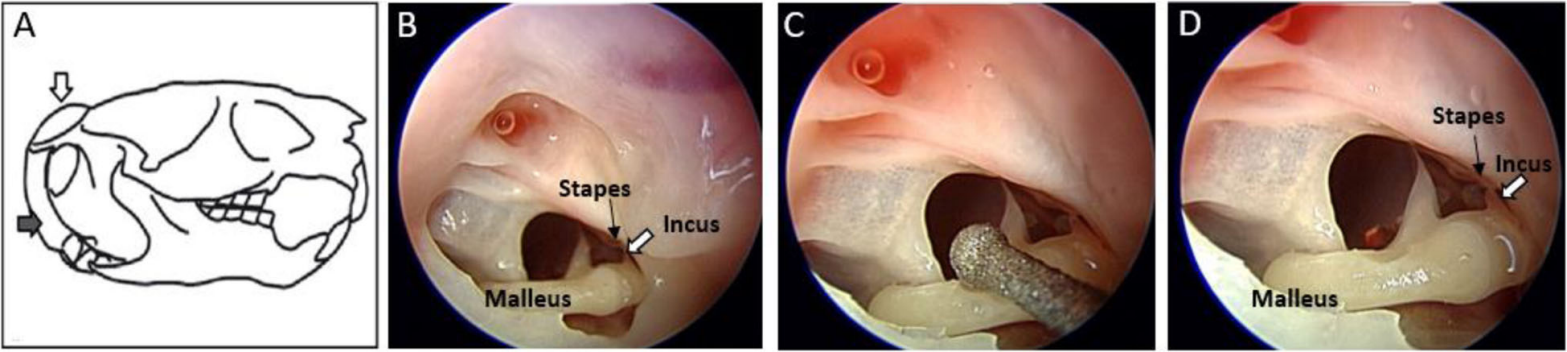
Surgical approaches to middle ear ossicles. **a** Schematic diagram of the skull of an adult *Chinchilla lanigera*. The malleus was accessed using a combination of a superior and inferior approach through the superior tympanic bulla (white arrow) and inferior tympanic bulla (gray arrow)*. **b** Initial view once the middle ear has been accessed via the inferior approach to the superior bulla with the malleus seen intact **(c)** with intervention using the tip of the ultrasonic bone removal device. **d** Small amount of bone removed from the malleus. Note *Diagram adapted for Wolter el. al [13]

The same surgical approach was carried out in each of the seven anesthetized adult chinchillas. A curvilinear incision using a #15 blade was made behind the test ear and carried to the level of the mandibular angle to expose the tympanic bullae. The superior bulla proper (Fig. 1, white arrow) was opened using a small rongeur, avoiding the venous sinuses, to expose the ossicular chain. The stapes footplate was kept in view during this maneuver, and if any movement was seen, the procedure was terminated. Meticulous hemostasis was maintained with bipolar diathermy to prevent bleeding into the middle ear.

For the purpose of conducting a dose-response analysis, the wound was closed and DPOAE measured after the following: opening the bulla, application of 10 s of UBD to malleus, and 2 subsequent re-application of 10s UBD in 4 ears. In the other 10 ears, the tip of the UBD was held against the body of the malleus for 15–30 s in order to remove a small amount of bone. Meticulous care was taken to ensure the incudostapedial joint remained intact with no disruption of the ossicular chain after this intervention. Minor bleeding that occurred was removed with suction or absorbed with tissue. No residual fluid was left in the middle ear cavity and clear hemostasis was ensured prior to wound closing. The ear canal and tympanic membrane were not disturbed by this approach to the middle ear.

For comparative purposes, surgical intervention was also attempted with a second group of three chinchillas, using a traditional surgical drill (Skeeter Oto-tool Drill, Medtronic, Minneapolis, Minnesota, USA) using a round, 1 mm diamond burr.

### Preparation for scanning Electron microscopy

Scanning electron microscopy was carried out in subjects to assess the morphology of the haircells using a well-established protocol [14–16]. The animals were perfused with fixative via a transcardial perfusion with saline followed by 2.5% glutaraldehyde in sodium cacodylate buffer (pH 7.4 fixative). The bullae were removed, and a hole was punched in the posterior bulla to remove the outer bone to expose the cochlea. The ossicles were removed from the oval window entrance and a 30G needle and 1-mL syringe was used to gently flush cochlea with fixative. The cochleas were then incubated in fixative overnight at 4 °C. The subsequent day, the cochleas were flushed twice with 2% buffered osmium tetroxide, then sequentially dehydrated in serial rinses of 35, 50, and 70% alcohol. Careful attention was paid to avoid exposure to the air once the dehydration steps began so as to avoid architectural damage of the haircells due to the evaporative process. In 70% alcohol, the bony components of the otic capsule were removed to reveal the haircells. The cochlea was then divided in to 3 sections for SEM: the apex, middle, and base. The samples were placed in a specialized cage and placed into a critical point dryer. Samples were rinsed three times in CO2 aqueous solution and maintained at a pressure of 800 psi. Next, pressure was increased to 1000-1500 psi and temperature up to 42 °C, and the samples were immersed in 100% ethanol and critical-point dried. Specimens were then mounted on adhesive carbon sheets, gold-sputtered, and viewed under a scanning microscopy (Hitachi 3400 s).

### Analysis of results

All identifying information was removed from the hearing test results and SEM images to mask the investigators regarding the animal as well as pre-postoperative status. Stata version 15.1 (Statacorp, College Station, TX) was used for all statistical analysis.

## Results

Eight animals fit our pre-operative ABR threshold criterion for inclusion. One was lost to anesthesia-related complications, but the remaining seven animals showed no signs of distress during surgery or at any time before sacrifice. Average total anesthesia time was 1 h, 30 min. Seven chinchillas underwent successful middle ear surgery with removal of bone from the malleus and complete postoperative DPOAE and ABR recording.

**Fig. 2:**
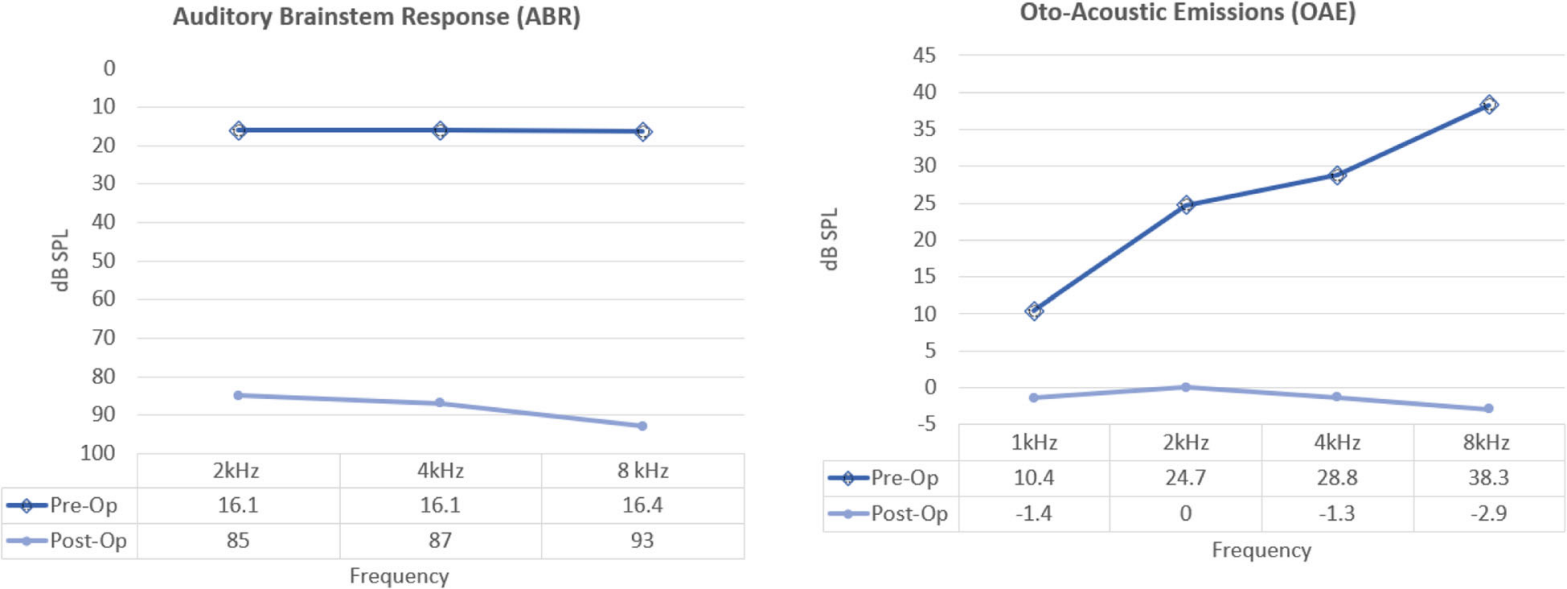
Preoperative and Postoperative ABR and DPOAE testing show profound hearing loss after surgical intervention. **a** Following surgical intervention with the UBD, there was profound hearing loss with an ABR threshold > 85 dB detected in 10 ears at 2, 4, and 8 kHz Average ABR threshold increased following intervention for all subjects: by 67 dB to 85 dB HL at 2 kHz (*p* < 0.05), by 71 dB to 8 dB HL7 at 4 kHz (*p* < 0.05), and by 77 dB to 83db HL at 8 kHz (*p* < 0.05). **b** In all of the animals, the OAE signal-to-noise ratio (SNR) was < 5 dB, consistent with profound hearing loss after intervention

As illustrated in Fig. 1, application of up to 30 s of UBD precisely removed a small quantity of bone from the malleus head without disruption of the ossicular chain or tympanic membrane. This procedure was duplicated in all ears. Pre and post surgical measures of auditory function are displayed in Fig. 2. The right panel plots average DPOAE signal-to-noise ratio (SNR) obtained before (open symbols) and after (closed symbols) removal of bone from the malleus. DPOAE SNR of < 5 dB SPL at 1, 2, 4, and 8 kHz, consistent with profound hearing loss occurred in all 14 ears after UBD intervention. Following intervention, DPOAE levels were reduced from pre-operative levels by an average of 12 dB (*p* < 0.05) at 1 kHz, 22 dB (p < 0.05) at 2 kHz, 29 (p < 0.05) at 4 kHz, and 42 dB (p < 0.05) at 8 kHz. As shown in the left panel of Fig. 2, following completion of the UBD intervention, ABR threshold elevations to > 85 dB SPL (closed symbols) were detected in 13 ears. Post-surgery, a clear ABR waveform was detected in only one ear, with thresholds of 40 dB at 2 kHz and 60 dB 4 kHz, but not at 8 kHz.

**Fig. 3:**
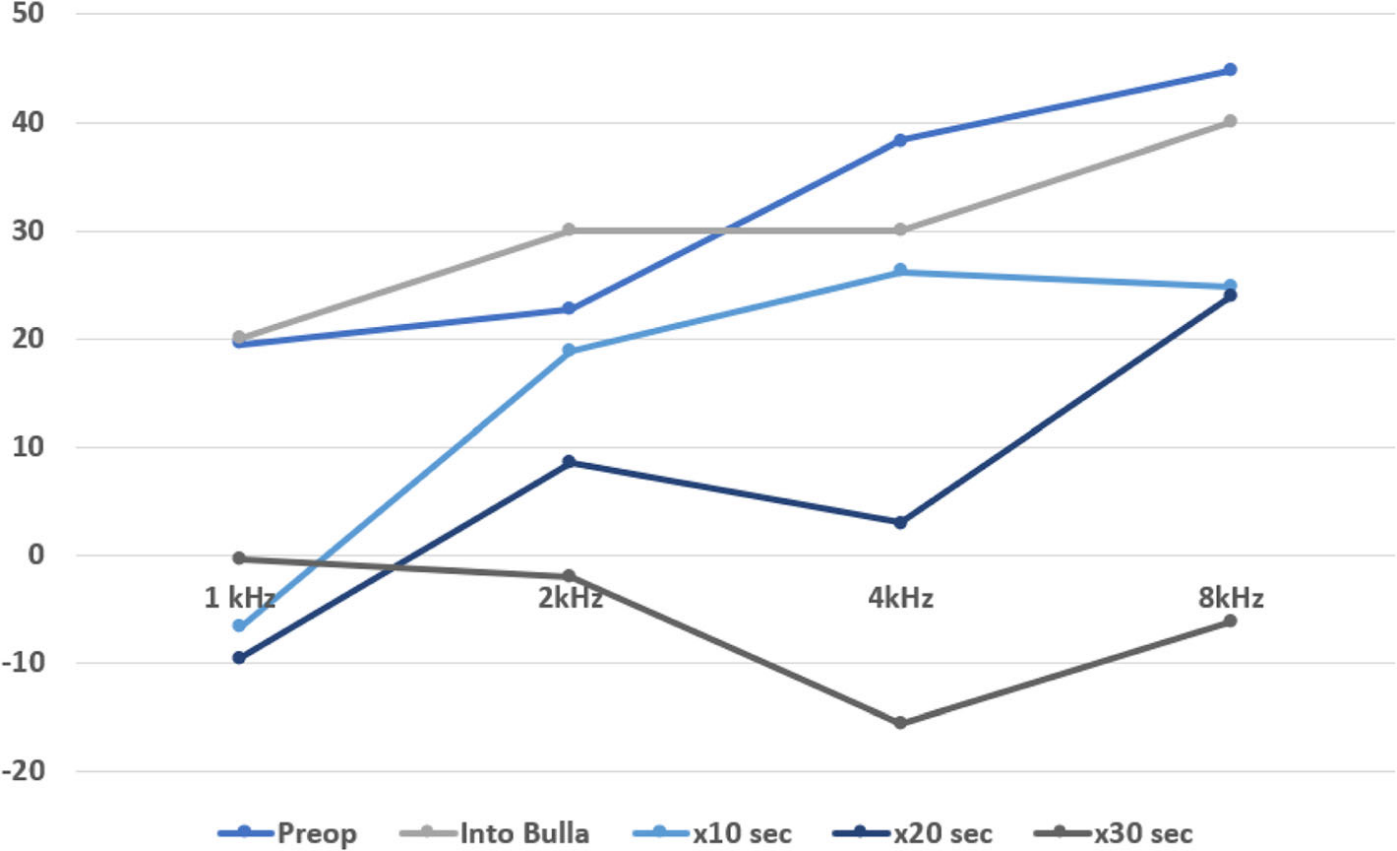
Dose Response Analysis. There was no difference in SNR after opening the bulla to gain access to the middle ear, however there was a trend towards a dose-response relationship with decreasing SNR after increasing applications of 10 s of UBD to malleus, and 2 subsequent re-application of 10s UBD

**Fig. 4:**
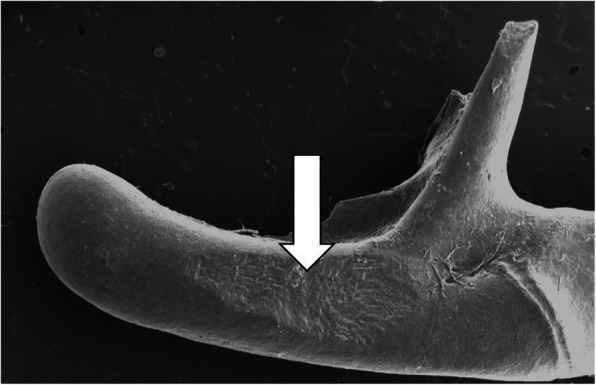
SEM of malleus showing the extent of bone removal after a 30s application of the with ultrasonic bone removal device

Figure 3 shows repeated measures of DPOAE SNR pre-op and then with increasing USD application times. There was no difference in SNR after opening the bulla to gain access to the middle ear. There is clear evidence of a dose-response with decreasing SNR after increasing applications of 10s of UBD to the malleus, up to 30s. Fig. 4 shows an example SEM micrograph of the extent of malleus bone removal after a 30s application of the UBD.

**Fig. 5:**
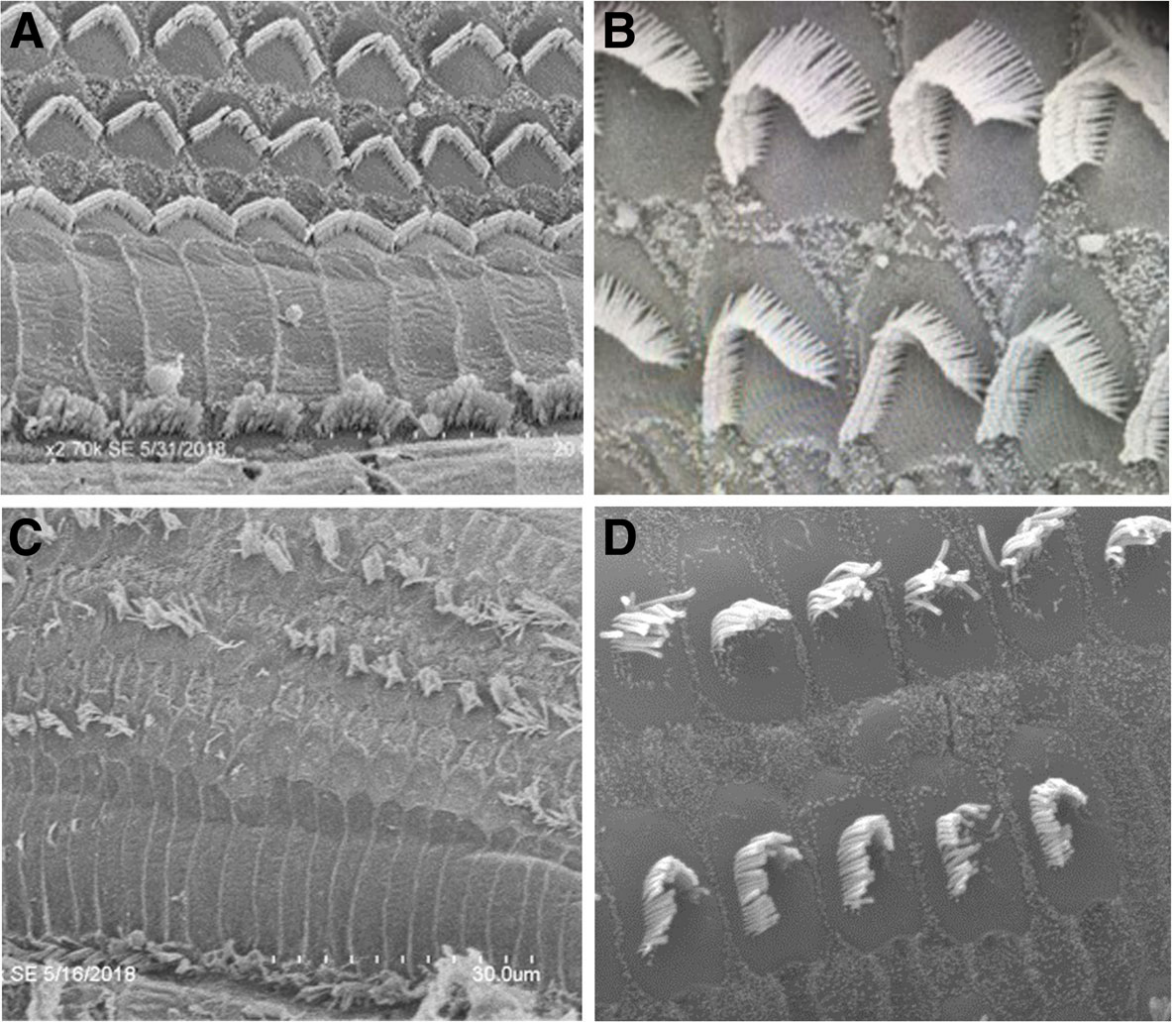
SEM micrographs of glutaraldehyde fixed, osmium tetroxide stained sensory epithelia specimens from chinchillas drilled with an ultrasonic bone drill and with traditional surgical drill. Panels **a** & **b** show typical SEM images of basal turn organ of Corti after malleus drilling with traditional surgical drill. There is little evidence of disruption of haircell structure and architecture. Panels **c** & **d** show representative images of the basal turn of organ of Corti after surgical intervention with the ultrasonic bone removal device showing destruction and distortion of haircells

Application of a diamond burr to the malleus head with the otologic drill at low power caused traumatic dislocation of the incudo-malleolar joint. It was not possible to use the drill to reshape the malleus in situ in this animal model. Figure 5 illustrates example SEM images of sensory epithelium in the basal cochlear turn in animals after drilling the malleus with the traditional surgical drill (panels A and B), compared with the ultrasonic device (panels C and E).

The cochleas after use of the traditional drill showed little evidence of disruption of haircell structure and architecture. In contrast, the cochleas after use of the ultrasonic device showed consistent disruption of outer haircell integrity.

## Discussion

It is widely recognised that application of an otologic drill to the ossicular chain can cause hearing loss. In guinea pig models, 10–60 s of drill-induced stimulus to ossicles has been shown to induce increased permeability of the capillary vessels of the stria vascularis [17, 18]. In a study by Paparella and colleagues cellular degeneration and severe disruption of the cytoarchitecture of the haircells in the organ of Corti at the basal turn was noted after drilling on the intact ossicular chain [19]. Similarly, Gjuric et al. also showed drill-induced damage to the inner ear after drill injury to the body of the incus. Caution when drilling around the ossicular chain to avoid sensorineural hearing loss was advised [20].

Given the limitations of drilling on an intact ossicular chain, a safer alternative would be beneficial for release of congenital ossicular fixation, or even for ossicular reshaping to access hidden cholesteatoma. The present study demonstrates that the UBD can precisely reshape an ossicle without visible injury to other structures of the middle ear, an impressive result given that the briefest touch with a delicate Skeeter drill was sufficient to dislocate the delicate ossicular chain in this model. However clinical utility of this intervention appears limited by the risk of inner injury as manifest by the significant reduction in DPOAE amplitude and increase in ABR threshold. This injury is greater at higher frequencies but is sufficiently severe to affect all frequencies and cause profound hearing loss. Shorter applications of the UBD to the malleus lead to a reduction in only high frequency OAEs. These findings indicate that more limited application to a mobile ossicular chain (such as could occur during inadvertent unintended contact during surgery) is likely to be significantly less damaging to the cochlea.

Our findings of both functional and structural damage after use of the UBD are consistent with the findings of some previous studies. Ultrasound ablation of the vestibule was used as a treatment for Meniere’s disease in the 1960s as developed and reviewed for example by Arslan [21]. His rabbit studies showed injury to the vestibular neuroepithelium. More than one third of patients experienced hearing loss in this and other reports [22] and 2% a facial nerve palsy. However, it is important to emphasize the difference in that old surgical technique and technology, where much higher frequency ultrasound (1-3 MHz) was applied for up to 30 min with probe temperatures reaching 40 °C. The UBD used in our study produces ultrasonic waves between 24.7–29.5 kHz, but, when using the same device near the inner ear, Cuda et al. found that patients experienced a significant deterioration of bone conduction at high frequency and postoperative vertigo [10, 11]. This occurred even when low-power settings were used for a small period of time for stapedotomy. Similarly, Pawlowski et al. detected significant trauma to the inner ear, manifested by basal turn haircell loss of rats who were subject to otic capsule drilling with the same UBD device [12]. However, other groups have reported no audiological or vestibular changes in the inner or middle ear of humans who underwent stapedotomy or otosclerosis surgery with the UBD device [5, 6, 23]. Other studies by Salami et al. have described use of the same UBD device in several otologic procedures including stapedotomy, attico-antrostomy, mastoidectomy, tympanotomy, facial nerve decompression, and excision of middle ear tumors [3–5, 23–25].

It is unclear exactly why these differences in outcome occur, however it is important to note the great degree of heterogeneity in the studies mentioned previously in regards to degree of intervention, the power level setting (varied from level 1 to level 7) and the degree of time the UBD was used in the middle ear (milliseconds to tens of seconds). Not surprisingly, higher power level used and longer duration trended towards showing greater damage. Notably there may be a dose-dependant relationship on clinical outcomes depending on the differential amount of energy delivered to the UBD tip with each of the different settings on the device. The device used in this study has the choice of bone anatomy type (Cortical, Cancellous Medium, Cancellous Low, and Delicate Anatomy), vibration level (continuous or spot, the latter of which activates the device for only 200 ms), and power level (1 to 7, refers to the power supplied to the cutting tip to generate the vibrational action with a tangential relationship to the energy at the actual tip). Notably, level 1 power has only 2% power supplied to the handpiece. Overall, evidence suggests that delivery of a larger amount of energy may have a significant effect on hearing outcomes in a clinical setting.

The loss of both ABR and OAE signals is consistent with significant damage to the inner ear, but the possibility of transient threshold shift cannot be excluded as recovery can occur over 3–7 days after injury [26]. Animals were euthanised after completion of surgical intervention in our protocol in accordance with Animal Care Committee guidance because of the complexity of providing appropriate supportive care after ablative surgery. Despite the possibility of a component of transient threshold shift by the study design, the magnitude of hearing loss makes recovery of normal hearing after this intervention unlikely. Furthermore, the morphological pattern of cochlear damage revealed by the SEM, specifically focal loss of outer haircells is most consistent with permanent sensorineural hearing loss [26]. A previous study reports this finding, showing similar focal loss of haircells and distinct structural changes with noise-induced permanent threshold shifts which are not present with temporary threshold shifts [27]. Further research is justifiable to further investigate the transient or permanent effect of ultrasonic bone removal.

There are several hypotheses proposed for inner ear trauma caused by ultrasonic bone drilling. The first theory proposed by Cuda, is that intense ultra-high-frequency sound generated by the device causes noise-induced trauma. In one of their studies, bone conduction deterioration occurred when the UBD was used for osteoplasty of the round window for middle ear implantation [10]. The second hypothesis is that inner ear trauma occurs after the generation of a shock-like pressure wave into the labyrinth fluids causing a cavitation effect with fluid displacement [10, 11].

Limitations to the current study include the use of an animal model with a normal ossicular chain upon which the effects of UBD may not be equivalent to those on human ear undergoing surgery. It is possible that the small skull of the chinchilla may alter its susceptibility to ultrasonic injury and that the current study over-estimates the damage caused by UBD. The damage may not be as severe as in humans and other animals. Indeed although the chinchilla has been used as an animal model for noise-induced hearing loss, there are other reports that in general, chinchillas are more susceptible to noise damage than many other species including humans and monkeys [28–30]. Regardless, there was still a striking the difference in inner ear structure between the UBD and the traditional drill. The effect of UBD on a fixed ossicular chain was not investigated with this model. As mentioned, any recovery from temporary threshold shift was not assessed. Also, the possibility of vestibular injury was not assessed. It is important to recognize that assessment of cochlear injury in humans after otologic surgery is difficult as OAE levels may be undetectable because of middle ear disease or older age, and bone conduction cannot be used for evaluation of high frequency hearing loss.

## Conclusion

Evidence from this study raises concern about the risk of cochlear injury from UBD with prolonged application of the device to the ossicular chain. The risk of sensorineural hearing loss from application of the device in close proximity to the cochlear should be considered. In the authors’ opinion, further study should be undertaken before consideration is given to use of the device for release of congenital or acquired ossicular fixation or to remove part of an ossicle to provide surgical access medial to a normal ossicular chain. Models of congenital ossicular fixation or otosclerosis and different UBD settings could be tested to validate this conclusion.

## Data Availability

All data and materials are available upon request.
